# Sex change in scallop *Patinopecten yessoensis*: response to population composition?

**DOI:** 10.7717/peerj.5240

**Published:** 2018-07-12

**Authors:** Alla V. Silina

**Affiliations:** Institute of Marine Biology, National Scientific Center of Marine Biology, Far Eastern Branch, Russian Academy of Sciences, Vladivostok, Russia

**Keywords:** Mollusks, Bivalves, Population, Scallops, Sex change, Protandry, Environment, Growth, Populations, Growth rates

## Abstract

Sex structure is very labile between populations and specific for each population because it is a result of genetic, ontogenetic and biocenotic influences on the mollusks. In this study, the age frequency distribution, age-sex structure, and sex ratio were assessed in the wild populations of the Yeso scallop *Patinopecten yessoensis* (Jay) observed at fifteen sites in the northwestern Sea of Japan (=East Sea). The sex ratio varied between the populations from 0.83:1 to 1.52:1 (males/females), with the mean sex ratio being 1.03 ± 0.05:1. Within a population, the proportions of males and females in term of number differed between age classes. Males were more numerous than females in the younger age classes, and females prevailed over males in the older age classes. It was found that in different scallop populations the sex change occurred at different ages. In the populations that predominantly consisted of young (two- to four-year-old) individuals, males prevailed over females in the age class 2 yr.; the equal male/female proportion was found in the age class 3 yr.; and in older age classes, females prevailed over males. Another pattern was observed in the populations that consisted mainly of middle-aged (five- to six-year-old) individuals. Here, the age-sex ratio became equal at an age of 4–6 years. In the old populations (mainly 6–12-year-olds) the equal male/female proportion was observed at an age of 8–10 years. Thus, the age of sex change was not uniform for the scallop populations. It depended on the age structure of the population and, thus, was socially controlled. The greater number of females in the older age classes suggests a protandric sex change.

## Introduction

It is known that among marine scallops the sequential production of oocytes and sperm occur in the same individual. However, the variations in the timing of sex change and the factors influencing the change remain poorly understood. One explanation is that the size at sex change is determined by environmental factors such as food abundance, chemical pollution, population density, and, as a consequence, growth rates (Hoch & Cahill, 2012; Merot & Collin, 2012a; Merot & Collin, 2012b). In marine mollusks, the timing of sex change is often labile and is thought to be largely influenced by interactions with conspecifics. Previous studies of calyptraeid gastropods and coralliophilid neogastropods showed that their social environment influences the timing of protandrous sex change (Richter & Luque, 2004; Dupont, Bernas & Viard, 2007).

The size advantage hypothesis predicts an optimal size at sex change, below which all individuals are of the first sex, and above which all individuals belong to the second sex. However, field studies commonly find a considerable overlap between male and female sizes and, therefore, fail to determine a population-wide optimal size for sex change (Wright, 1989; Soong & Chen, 1991; Sewell, 1994). The variation in size at sex change, resulting in the overlap between the male and female sizes, is explained by the fact that animals change sex based on cues like age, which may not correlate exactly with size, and the same-sized bivalves may have different ages. The lack of a method to determine individual age, especially for specimens from wild habitats, makes it impossible to compare the results for different populations and same-aged mollusks.

For the Yeso scallop *Patinopecten (*=*Mizuhopecten) yessoensis* (Jay, 1857), there is a way to assess the sex ratio for each age group in the wild population, as the author of the present study earlier developed a method of age determination and retrospective assessment of shell height growth for each scallop specimen during its lifecycle (Silina, 1978; Silina, 1996). The method is based on the growth layers in the external surface microsculpture of the upper valve of scallop. The winter and summer growth layers differ in their appearance and width. Scallop forms one broad elementary growth layer weekly during November–April and one narrow elementary growth layer daily during the rest of the year. The visible thickening of the narrowest layers (ring) occurs annually, in August. This allows determination of both the age of scallop, by counting the number of annual shell rings, and its individual linear growth rates in retrospect, by measuring the heights of scallop shell from its apex to each annual ring. Yeso scallop is a suitable species to investigate the age-related features of sex change in bivalve populations.

Yeso scallop is common to subtidal habitats along the coasts of the Sea of Japan (=East Sea) and the Sea of Okhotsk. This scallop is a large-sized (up to 220 mm in shell height), long-lived (reaching a maximum age of 22 years), and mobile species (Silina, 1990). At 1 year of age, all individuals of this species are hermaphrodites, which do not spawn yet (Yamamoto, 1964; Mori, Osanai & Sato, 1977). In the study area, scallops reach sexual maturity at two years of age and start to perform the male or female functions. From this age, scallops become dioecious, with a low incidence of hermaphrodites (Yamamoto, 1964; Bregman, 1979). Spawning occurs once a year; the spawning season can vary depending on the environment but is usually limited to 1–1.5 months during spring and summer (Yamamoto, 1964; Mori, Osanai & Sato, 1977; Dzyuba, 1986; Chung et al., 2005). The species is presumably protandric (Yamamoto, 1964; Mori, Osanai & Sato, 1977; Maru, 1978; Otani et al., 2017). Fertilization is external, with sperm and eggs cells released into the sea water. Yeso scallop continues to grow after attaining sexual maturity. Its reproductive investments co-vary with age (Silina, 2016).

As Yeso scallop is commercially valuable seafood species, widely farmed in marine aquaculture, it is necessary to understand the sex structure of its populations to develop a proper management strategy. The purpose of the present study was to identify the social factors influencing the age of sex change in Yeso scallop by determining the sex ratio, the age frequency distribution, and the age-sex structure of each population and by comparing the variations in age of sex change between different wild scallop populations.

## Materials and Methods

**Figure 1:**
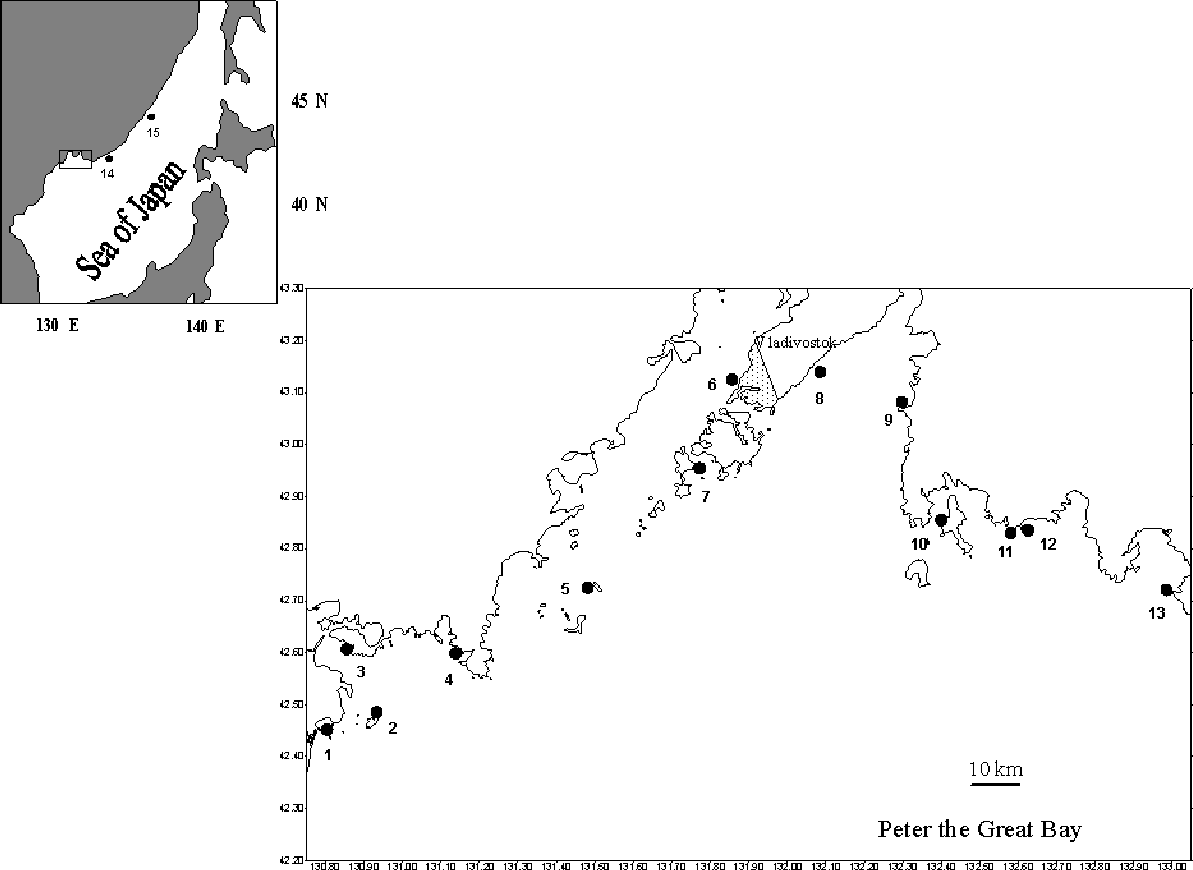
Sites of sampling of the scallop *Patinopecten yessoensis* (black circles). 1, Sivuchiya Bay; 2, Severnaya Bay; 3, Kreiserok Bay; 4, Vityaz Bay; 5, near Stenina Island; 6, Pervaya Rechka River Estuary; 7, near Cape Shkota; 8, Lazurnaya Bay; 9, Andreeva Bay; 10, near Putyatina Island; 11, Anna Bay; 12, Rifovaya Bay; 13, Ozero Vtoroe Bay; 14, Melkovodnaya Bay; 15, Srednyaya Bay.

Yeso scallops were collected by SCUBA divers at fifteen sites in the northwestern Sea of Japan (=East Sea), located mainly in Peter the Great Bay, in 1974–2016 (Fig. 1). Only wild scallop populations were studied. The field experiments were approved by the National Scientific Center of Marine Biology, Far Eastern Branch, Russian Academy of Sciences. At fourteen sites with quite clean water, the scallop samples were collected once during the study period (Table 1). At the polluted site, in the vicinity of the city of Vladivostok, in the Pervaya Rechka River Estuary (Site 6, Fig. 1), sampling was conducted twice, in 1994 and 2016. Each sample included 31–209 individuals (Table 1). Scallops were collected in May–June, shortly before or immediately at the onset of spawning, when they had mature gonads, and the sex could be identified visually by the gonad color. The gonad in female Yeso scallop is pink, and the male gonad is creamy (Yamamoto, 1964; Mori, Osanai & Sato, 1977; Dzyuba, 1986). For rare hermaphroditic individuals with bicolored gonads, sex was identified by the presence of sperm and egg cells under a binocular microscope.

**Table 1:** *Patinopecten yessoensis*. The male:female ratios in the wild scallop populations inhabited the northwestern part of the Sea of Japan. The populations are arranged according to increase in their most abundant age classes.

Site, year of sampling	Total number, in parenthesis are numbers of scallops at the age ≤5 and > 5 years old	Age range, years	Most abundant age classes, years	Male:female ratio for population	Starting age of female prevalence, years	Male:female ratio for age classes ≤ 5 years	Male:female ratio for age classes > 5 years
Anna Bay, 1988	59 (55 + 4)	2–7	2	1.36:1	4	1.62:1	**–**
Pervaya Rechka River Estuary, 2016	44 (44 + 0)	2–5	2 and 4	1:1	3	1:1	**–**
At Cape Shkota, 1979	37 (21 + 16)	2–10	2 and 7	1.06:1	4	2:1	**0.45:1**
Srednyaya Bay, 1988	73 (44+29)	2–12	3 and 4	1.52:1	4	1.87:1	1:1
Pervaya Rechka River Estuary, 1994	74 (49+25)	2–10	3 and 6	1:1	4–5	1.13:1	**0.79:1**
At Putyatina Island, 1980	46 (31 + 15)	2–11	4	1:1	6	1.58:1	**0.36:1**
**Rifovaya Bay, 1974**	50 (37 + 13)	3–9	4 and 5	**0.85:1**	5	1.06:1	**0.44:1**
Melkovodnaya Bay, 1980	61 (33 + 28)	1–14	5	1:1	6	1.64:1	**0.61:1**
Ozero Vtoroe Bay, 1980	209 (99 + 110)	1–10	5 and 6	1:1	5	1.36:1	**0.77:1**
Lazurnaya Bay, 1980	40 (12 + 28)	3–14	4 and 11–12	1:1	10	5:1	**0.56:1**
**Kreiserok Bay, 1996**	25 (7 + 18)	3–9	6	1.08:1	5–6	1.33:1	1:1
Andreeva Bay, 1980	68 (28 + 40)	2–13	2 and 6–8	**0.89:1**	6	3:1	**0.38:1**
**Sivuchiya Bay, 1996**	34 (10 + 24)	4–8	7 and 5	**0.83:1**	6	2:1	**0.60:1**
Vityaz Bay, 1979	41 (3+38)	4–13	8	**0.95:1**	9	–	**0.95:1**
**At Stenina Island, 1979**	31 (2 + 29)	4–16	9 and 12	1:1	11	–	1.07:1
**Severnaya Bay, 1999**	102 (15 + 87)	3–14	11	**0.87:1**	8	1.80:1	**0.78:1**

**Notes.**

Bold ratios indicate the higher proportion of females.

Bold and underlined names of the sites indicate populations, where the scallops have the high and low growth rates, correspondingly. An “–” denotes too little data for corresponding index calculation.

Age of each scallop was determined by counting the number of annual shell rings (Silina, 1978; Silina, 1996). Individual linear growth rates were assessed in retrospect by measuring the heights (dorso-ventral axis) of scallop shell from its apex to every annual ring. Sex ratio, age frequency distribution, and age-sex structure were calculated for each population.

Mean ± SEM (standard error of the mean) values of scallop shell heights in were calculated for each age in each population. A *t*-test was applied to reveal differences in mean value of shell height at each age between the populations. Prior to statistical analysis, all data were tested for normality of variances using a Kolmogorov–Smirnov test.

## Results

It was found that the wild populations of Yeso scallop differed in age frequency distribution, as well as in sex ratio. The study revealed a variability of sex ratio between the populations, from 0.83:1 to 1.52:1 (males/females), with the mean sex ratio being 1.03 ± 0.05:1. In the young populations (predominantly consisting of two- to four-year-old individuals), males usually prevailed, and the sex ratio ranged from 1:1 to 1.52:1 (mean, 1.19 ± 0.11:1) (Table 1). However, the old populations (mainly six to 11-year-olds) were dominated by females, and the sex ratio was from 0.83:1 to 1:1 (mean, 0.91 ± 0.02:1) (Table 1).

**Figure 2:**
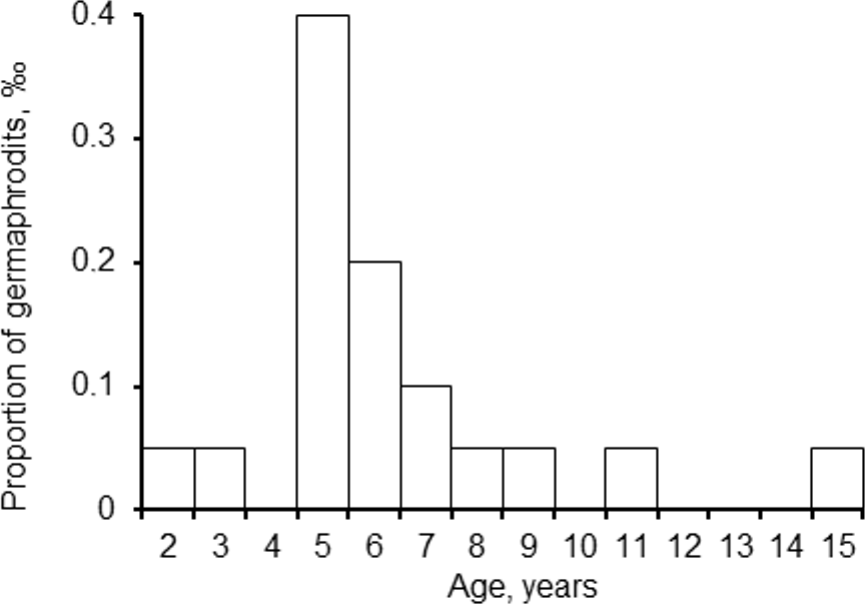
*Patinopecten yessoensis*. The proportion of different aged hermaphrodites per 1 thousand scallops at ages ≥ 2 years.

The frequency of occurrence of hermaphrodites was usually low, 0–2 ind. per hundred scallops aged ≥ 2 years. Only in the population inhabiting Ozero Vtoroe Bay (Site 13, Fig. 1), five hermaphrodites were found among 209 sampled scallops, which was a little higher than an usual value. The individuals with both male and female gametes in their gonads were from two to 15 years of age (Fig. 2). The maximal occurrence of hermaphrodites was among five-year-old individuals (Fig. 2). Usually, hermaphroditic gonads were creamy with small pink dots. Only one specimen had the pink female gonad with creamy dots.

The analysis of sex ratio in different age classes within each population indicated that males were more numerous than females in the younger age classes, and, *vice versa*, females prevailed over males in the older age classes, with significantly more females occurring in the oldest age classes among all studied populations (Table 1).

**Figure 3:**
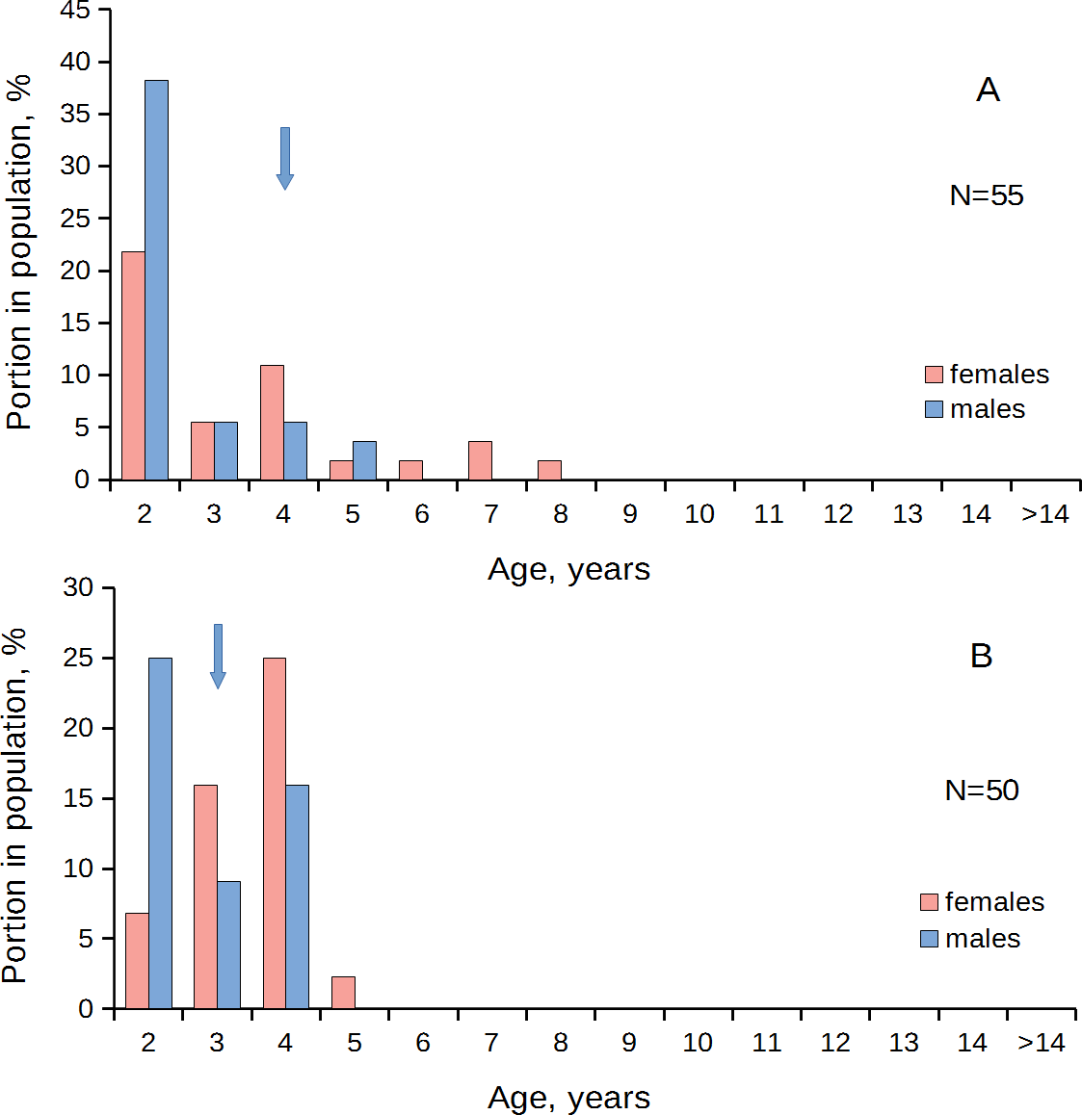
*Patinopecten yessoensis*. Sex-age structure of “young” populations (mainly two- to five-year-olds) from Anna Bay (A) and Pervaya Rechka River Estuary (2016) (B). Arrow indicates the first age class in which females start to prevail over males in number.

**Figure 4:**
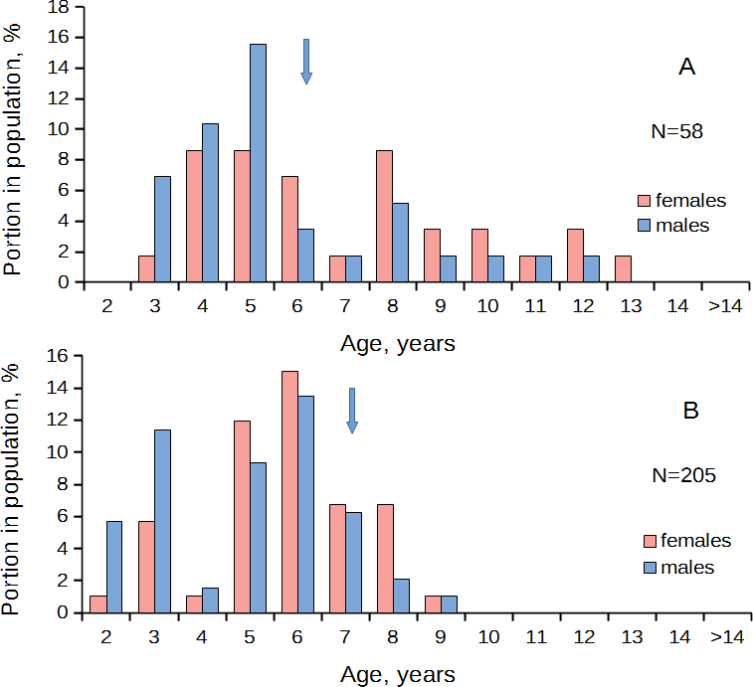
*Patinopecten yessoensis*. Sex-age structure of “middle aged” populations (mainly four- to eight-year-olds) from Melkovodnaya Bay (A) and Ozero Vtoroe Bay (B). Arrow indicates the first age class in which females start to prevail over males in number.

**Figure 5:**
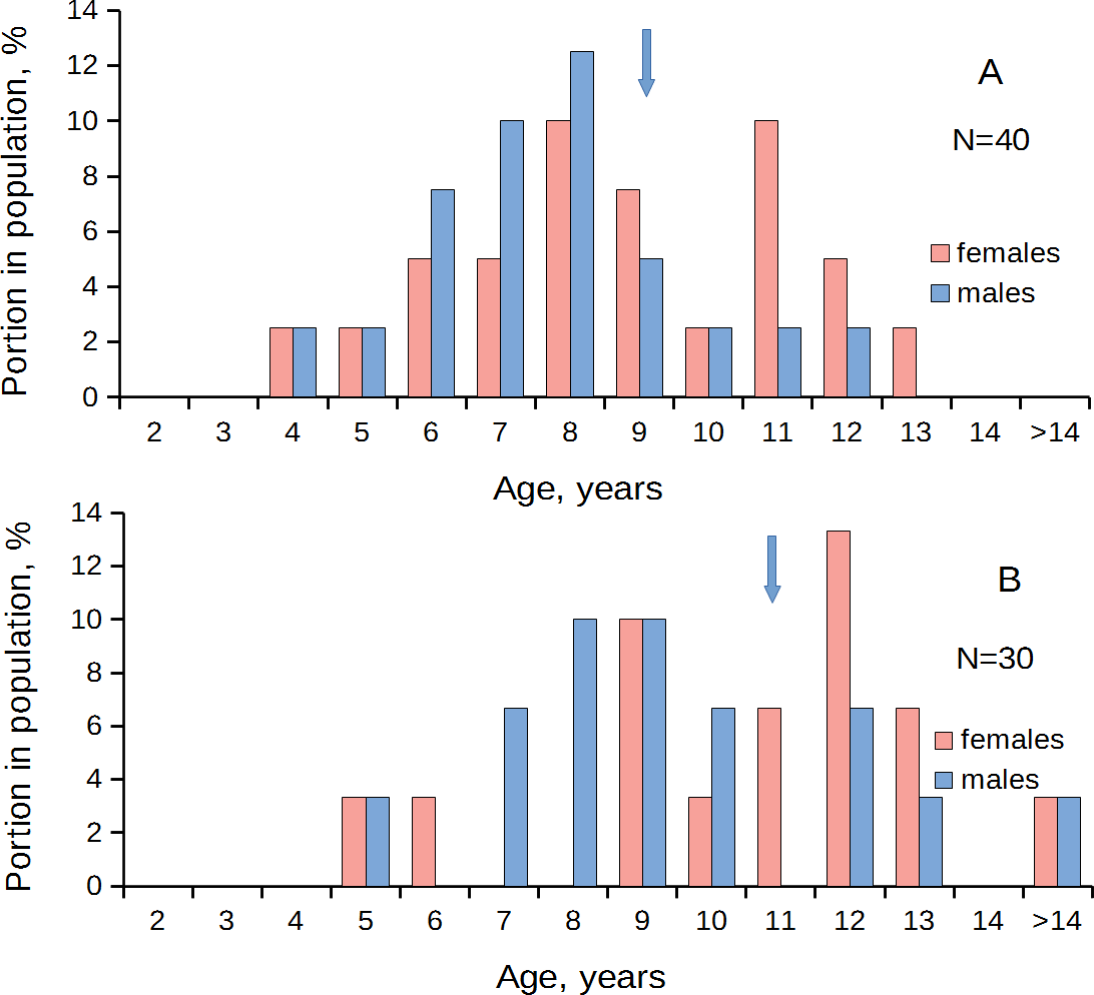
*Patinopecten yessoensis*. Sex-age structure of “old” populations (mainly six to 12-year-olds) from Vityaz Bay (A) and near Stenina Island (B). Arrow indicates the first age class in which females start to prevail over males in number.

In the populations that predominantly consisted of young (two- to four-year-old) individuals, males prevailed over females in the age class 2 yr.; the equal ratio was found in the age class 3 yr.; and in the older age classes females prevailed over males (Fig. 3, Table 1). A different pattern was observed in the populations that consisted mainly of middle-aged (4–6-year-old) individuals. Here, the sex ratio became equal at the age of four to six years (Fig. 4, Table 1). In the old populations (mainly six to 12-year-olds), the equal sex ratio was observed at the age of eight to nine years (Fig. 5, Table 1). Everywhere, the male/female ratio for each age in the populations decreased in the older age classes. Thus, no standard optimal age of sex change can be determined for all scallop populations. Males change the sex earlier in the presence of smaller males (compare Figs. 3–5).

As the environmental factors may influence on mollusks’ sexual maturation, the scallop growth rate was used as an integral index of environment quality for the species. The growth rates of the scallops collected from different sites were compared. The studied populations were divided into groups with low, middle and high growth rates (Table 2). The division into groups was carried out according to results of *t*-test of the shell heights of the same-aged scallops compared between the populations. If the shell heights were significantly (*P* < 0.05) lower than ones in the other populations for three or more age classes, this population was referred to the group with low growth rates. Similarly, the populations, in which the scallop shell heights were significantly (*P* < 0.05) higher than ones in the other populations for three or more age classes, were referred to the group with high growth rates. The remaining populations formed the group with middle growth rates. No relationship was found between the scallop growth rates and the age when females started to prevail over males in the population (Table 1). Scallops from the group with low growth rates could start to change sex later than ones from the group with high growth rates, for instance, in Andreeva Bay (Tables 1 and 2). And, *vice versa,* under optimal environment conditions the scallops with a high growth rates could change sex at a young age, for example, in Rifovaya Bay (Tables 1 and 2).

**Table 2:** *Patinopecten yessoensis*. The densities of the occurrences and growth rates in the populations inhabited the northwestern part of the Sea of Japan.

Site	Density, ind./m^2^, at site of sampling	Shell height at 1-year-old age, mm	Shell height at 2-year-old age, mm	Shell height at 3-year-old age, mm	Shell height at 4-year-old age, mm	Shell height at 5-year-old age, mm
		Low	Growth	Rates		
Srednyaya Bay	0.29	40.1 ± 2.6	79.1 ± 2.8	97.9 ± 2.4	116.4 ± 2.8	130.3 ± 2.8
Pervaya Rechka River Estuary	0.13	38.1 ± 0.7	73.5 ± 1.5	95.6 ± 1.5	108.3 ± 1.5	113.4 ± 1.0
Ozero Vtoroe Bay	0.80	42.4 ± 0.6	81.0 ± 0.7	104.7 ± 0.6	121.3 ± 0.7	132.5 ± 1.1
Lazurnaya Bay	0.16	43.4 ± 1.9	80.6 ± 2.3	104.5 ± 2.2	121.5 ± 2.3	127.5 ± 1.7
Andreeva Bay	0.31	48.6 ± 0.8	88.7 ± 1.0	108.0 ± 1.0	119.8 ± 1.1	125.6 ± 1.3
		Middle	Growth	Rates		
Anna Bay	2.01	52.5 ± 0.8	86.9 ± 0.9	121.2 ± 0.7	134.4 ± 1.1	138.2 ± 1.8
At Cape Shkota	0.19	48.3 ± 2.1	89.5 ± 1.7	116.4 ± 1.3	129.2 ± 1.4	133.6 ± 1.9
At Putyatina Island	0.16	50.2 ± 1.6	87.4 ± 1.2	117.0 ± 1.0	129.1 ± 1.1	135.3 ± 1.8
Melkovodnaya Bay	0.25	39.5 ± 1.8	81.7 ± 1.3	108.8 ± 1.4	126.3 ± 1.3	137.7 ± 1.5
Vityaz Bay	0.27	47.2 ± 1.3	89.5 ± 1.1	116.1 ± 0.9	130.6 ± 1.1	140.0 ± 1.5
		High	Growth	Rates		
**Rifovaya Bay**	0.60	45.6 ± 1.8	96.1 ± 2.0	118.7 ± 1.8	137.2 ± 1.5	148.6 ± 2.3
**Kreiserok Bay**	0.22	62.6 ± 1.4	106.2 ± 1.3	129.0 ± 1.3	141.8 ± 1.4	149.4 ± 1.2
**Sivuchiya Bay**	1.03	65.4 ± 1.0	108.4 ± 0.9	131.2 ± 0.8	143.1 ± 0.8	151.3 ± 0.8
**At Stenina Island**	0.24	45.0 ± 1.2	89.4 ± 1.5	120.5 ± 1.4	138.5 ± 1.6	150.4 ± 2.3
**Severnaya Bay**	0.67	52.7 ± 1.2	94.3 ± 1.2	116.2 ± 1.3	130.0 ± 1.9	138.8 ± 1.9

**Notes.**

Bold and underlined names of the sites indicate populations, where the scallops have the high and low growth rates, correspondingly.

At the sampling sites, the density of scallop distribution varied widely, from 2.01 to 0.13 ind./m^2^ (Table 2). No relationship has been found between the density of scallop distribution and the age when the females started to prevail over males. In the group with low scallop growth rates, the density of scallop distribution varied from 0.80 to 0.13 ind./m^2^ (mean, 0.34 ± 0.12); in the group with middle growth rates, it was from 2.01 to 0.16 ind./m^2^ (mean, 0.58 ± 0.35); in the group with a high growth rates, it varied from 1.02 to 0.22 ind./m^2^ (mean, 0.56 ± 0.15). The differences between the mean values were not statistically significant (*P* > 0.05).

## Discussion

In general, the sex ratio in the Yeso scallop populations does not considerably differ from 1:1, being slightly male-biased. In some scallop populations, the sex ratio is skewed toward females, and in others it may be skewed toward males. The above finding agrees with what was reported for this scallop species earlier, for example, by Maru (1978) and Bregman (1979), who found generally a 1:1 sex ratio for wild Yeso scallop populations. However, in the present work, it has been found that the young age classes show strongly male-biased sex ratios in each population, and females become increasingly more prevalent with age. The greater number of females in the older age classes suggests a protandric sex change (Bauer, 2000). This conclusion for Yeso scallop is consistent with the assumption of Yamamoto (1964), Mori with coauthors (1977), Maru (1978), and Otani with coauthors (2017) that Yeso scallop is protandrous (i.e., sex changes from male to female). It is known that among the eight molluscan classes only the Gastropoda and Bivalvia have sex-changing species, of which almost all are protandrous (Wright, 1988). Our case is not a strict protandry, as all one-year-old individuals of this species are hermaphrodites (Yamamoto, 1964; Mori, Osanai & Sato, 1977; Maru, 1978; Otani et al., 2017), and subsequently a small percentage of scallops first become females without any preceding male phase. In a truly protandric species, all individuals in population mature first as males and then become females when they increase in size and age (Bauer, 2000). Protandric populations have their size-frequency distribution with a relatively small overlap between the male and female phases, which is confirmed by an observation of transitional individuals having both male and female characters at an intermediate size (Bauer & Newman, 2004). In Yeso scallop populations, there are transitional individuals having both male and female characters in a wide age range.

Protandry is a widespread sexual strategy. It is expected in cases when female egg production increases with age, while the male’s ability to fertilize eggs is practically independent of age (Warner, Robertson & Leigh, 1975; Warner, Fitch & Standish, 1996). Earlier, it was found that Yeso scallop has marked differences between age classes in the reproductive investment: younger scallops have a lower reproductive output than older individuals (Silina, 2016). Besides, a statistically significant female-biased dimorphism in the gonad weight was revealed for the scallop age classes >4 yr. The values of shell growth rate and size in both sexes were similar (Silina, 2016). Therefore, for large-sized individuals that have large gonads, it is advantageous to change sex from male to female in order to maximize the reproductive success of population. The fecundity advantage hypothesis for Yeso scallop, with its group mating and external fertilization, is at least partly implemented by the physiological mechanisms, which provide larger gonads for older females than for older males in a population with the aim to produce a larger brood. Gregarious settlement of this bivalve (Silina & Bregman, 1986) contributes to the reproductive success of population, as the energetically costly ovaries may all be fertilized by sperm of small males in a scallop aggregation.

The age of sex change in Yeso scallop is not uniform; it depends on the age structure of a population and thus is socially controlled. It is known that sex change in many hermaphrodite animals is often labile and assumed to be environmentally determined (Dupont, Bernas & Viard, 2007). The plasticity in age of sex change suggests a socially control of sex change. A protandrous hermaphrodite is strongly influenced by the composition of the local group (Dupont et al., 2006; Dupont, Bernas & Viard, 2007). The studied protandrous scallop species has the potential to change sex from male to female depending not only on age, but also on the local social environment. In Yeso scallop populations, males change their sex earlier in the presence of smaller males, which can support successful egg fertilization. This mechanism may adjust the reproduction success in different generations (Dupont, Bernas & Viard, 2007).

It may be expected that the variations in the age of sex change across the populations result from the genetic distinction of the scallop populations. However, earlier, the genetic structure of Yeso scallop populations from southern Primorsky Krai, Russia, (studied area) was investigated using four microsatellite markers and a mitochondrial marker, and little evidence of divergence within this region was found (Sato et al., 2005). It was revealed that the scallop samples from Dalian (China) and Japan are closely related, while the population from Russia forms a distinct clade in the phylogenetic analysis (Liu et al., 2010). Also, it was shown that a large, genetically homogenous population inhabits the study area of the Sea of Japan, except for the population at the Site 2, Severnaya Bay (Site 2, Fig. 1), which is close to the Korean population (Dolganov & Pudovkin, 1997). However, in the present work, the latter population did not show any specific feature. The results of the study of scallops from this population agree in general with the obtained data.

It is known that pollution and other adverse environmental factors may affect sexual maturation of mollusks (Hoch & Cahill, 2012; Merot & Collin, 2012a; Merot & Collin, 2012b). In the study area, only the Pervaya Rechka River Estuary (Site 6, Fig. 1) is polluted, as it is located on the coasts of Vladivostok City (Tkalin, Belan & Shapovalov, 1993; Shulkin & Kavun, 1995). Indeed, the growth rates of scallops are low here (Table 2). However, in 1994, when the degree of pollution was the highest for the study period (Lukyanova, Cherkashin & Simokon, 2012), the population characteristics at this Site were usual. Sex ratio was 1:1, and the age when females start to prevail over males was four to five years (Table 1). Later, the anthropogenic impact on the benthos decreased due to the decline in industrial activity (Lukyanova, Cherkashin & Simokon, 2012). Nevertheless, in 2016, the population characteristics changed due to scallop harvesting. The sex ratio remained the same, but the age classes ranged from two to five years in this population, and females started to prevail already among three-year-olds (Table 1). The peculiarities revealed in the present work are expressed most pronouncedly under such environmental conditions. There were no particularly adverse conditions for Yeso scallop at the other study sites.

The differences in growth rate between the scallop populations are explained by variations in the key environmental conditions at the study sites. Growth rate is a cumulative indicator of environment quality for this species. Yeso scallop is a stenohaline species; it prefers a high oxygen content of water, various-grained sand with a silt admixture, and a water temperature of 4–20 °C (Silina, 1990). Indeed, the highest growth rates were observed in open bays with good water aeration, and minor short-term seasonal decreases in water salinity. All the sites of the selected groups with high growth rates (Table 2) have the environmental parameters close to optimal for scallop. The groups of populations with low scallop growth rates were from the sites with mostly suboptimal environmental conditions. At the present work, it was found that the age at which females start to prevail over males in the population is not determined by growth rates. However, most likely, at the sites with conditions favorable for scallop the probability of longer life cycle is high. Therefore, the populations found at such sites are often, but not always, dominated by the age classes >5 yr. (Table 1).

It is known, that starvation may interfere with sexual maturation (Merot & Collin, 2012a; Merot & Collin, 2012b). Starvation of Yeso scallop in wild populations from the study area has never been mentioned in a literature. This scallop feeds on a mixed, but diatom-dominated phytoplankton diet (Silina & Zhukova, 2009). According to some estimates, the diatom genera *Thalassiosira, Coscinodiscus, Pseudo-nitzschia, Chaetoceros, Thalassionema, Rhizosolenia,* and *Skeletonema* account for about 60–90% of the total phytoplankton biomass in the study area. Their biomass is especially high during winter and spring, when scallops’ gonads mature most intensively (Stonik & Selina, 1995; Aleksanin et al., 2012).

The aquatic lifestyle of Yeso scallop has important implications for chemical communication. The multiple and diverse chemosensory structures found in animals of this species provide evidence that they continuously scan their environment for chemical information (Khotimchenko & Deridovich, 1991; Deridovich & Reunova, 1993; Tanabe et al., 2010). Chemical cues have gained overriding importance. It is known that many mollusks perform sex change, and sex hormones may be involved in the process (Otani et al., 2017). Steroids, such as estradiol and testosterone, are involved in sexual differentiation and gonad development (Wang & Croll, 2004). Injections of estradiol, testosterone, progesterone and dehydroepiandrosterone significantly promoted sexual differentiation and shifted sex ratios, resulting in more males than females, as well as causing other morphological changes in scallops (Croll & Wang, 2007). It is known that serotonin and dopamine levels are higher in the male gonadal portion than in the female portion in the scallop *Argopecten purpuratus*, when gametogenesis is proceeding (Martinez & Rivera, 1994). The present work has shown that scallops change sex in the populations with various densities of distribution, and, thus, the determinative factor for sex change is most likely a certain ratio of biochemical substances in the sea water rather than the absolute values of their concentrations. Besides, it is known that some substances may enhance the sensitivity of Yeso scallop gonad to external stimuli during progressing maturity (Osada, Mori & Nomura, 1992).

## Conclusion

The young age classes have a male-biased sex ratio in each Yeso scallop population, and females become more prevalent as the age increases. This suggests a protandric sex change. This means that, for Yeso scallop, it is advantageous to change sex just from male to female in order to maximize the population reproductive success, as the female’s egg production increases with age throughout the life cycle (Silina, 2016), and the male’s ability to fertilize eggs is practically independent of age.

The age of sex change in Yeso scallop depends on the age structure of the population and thus is socially controlled. In populations, males change sex earlier in the presence of smaller males, which can support successful egg fertilization.

##  Supplemental Information

10.7717/peerj.5240/supp-1 Data S1 Age, total shell height, sex, and shell heights at each age of Yeso scallops from different populations

## Additional Information and Declarations

### Field Study Permissions

The following information was supplied relating to field study approvals (i.e., approving body and any reference numbers): Field experiments were approved by the National Scientific Center of Marine Biology, Far Eastern Branch, Russian Academy of Sciences in 1974–2016.

### Data Availability

The following information was supplied regarding data availability: The raw data are provided in Data S1.
